# Somatization in dermatology

**DOI:** 10.1002/ski2.164

**Published:** 2022-09-29

**Authors:** George W. M. Millington, Morinola T. Shobajo, James Wall, Mohammad Jafferany

**Affiliations:** ^1^ Norwich Medical School Norwich UK; ^2^ Dermatology Department Norfolk and Norwich University Hospital Norwich UK; ^3^ Department of Dermatology University of Illinois at Chicago College of Medicine Chicago Illinois USA; ^4^ Department of Psychiatry Central Michigan University/CMU Medical Education Partners Saginaw Michigan USA

## Abstract

Medically unexplained dermatologic symptoms, such as pruritus, numbness and burning are known as somatization. These cutaneous symptoms can be very difficult to treat because of an absence of an objective explanation and they may not fit neatly into any known dermatological or psychiatric condition. These disorders are more commonly encountered in primary care and in dermatology, rather than in psychiatry. Certain skin disorders, for example, pruritus, could be a manifestation of somatization and others may predispose to somatic symptoms, for example, atopic dermatitis and psoriasis. Although there has been increasing research in the interconnection between psychiatry and dermatology, psychodermatology is a relatively new crossover discipline in clinical practice and recognition of psychodermatological conditions, such as cutaneous somatic disorders, can be difficult. Somatization may occur with or without the existence of a dermatological disease. When a dermatological disorder is present, somatization should be considered when the patient is worrying too much about their skin, spending too much time and energy on it and especially if the patient also complains of many non‐cutaneous symptoms. Purely cutaneous somatic conditions include for example, the genital pain syndromes or Gardner–Diamond syndrome, characterized by unexplained bruising, which usually affects women. Effective management tools may include mindfulness therapies, pharmacotherapy with selective serotonin reuptake inhibitors, tricyclic antidepressants and cognitive conduct therapy. Electroconvulsive therapy can also be considered in extremely rare cases for treatment of severe somatization on a background of mood disorders. This paper discusses somatization, its relationship to immunodermatoses and its relevance to clinical practice.

1

**What’s already known about this topic?**

- It is well known that psychological disorders can mimic skin disease. Equally skin disease is frequently associated with psychiatric and psychological disorders.

**What does this study add?**

- This study defines a framework for somatisation in Dermatology. This is within the DSM Classification of diseases.

## 2 INTRODUCTION

Somatization is defined as a symptom that results from an underlying psychological disorder, distress, or early life trauma.^1^ The skin is the primary organ of attachment in early life and used for communication.^1^, ^2^ Therefore, it may be ‘vulnerable’ to the development of somatization.^1^, ^2^ A common derivative of somatization can stem from psychological trauma and/or neglect during early life.^1^, ^2^ The most common symptoms presented are pruritus, numbness, burning, soreness, and blotchiness (Table 1).^1^, ^3^ These more commonly involve women.^1^, ^3^ These symptoms are often localized to the face, scalp and perineal areas.^2^ Medically, the symptoms are usually unexplained from a dermatological standpoint.^2^ The skin is a sensory organ, which responds to emotional stimuli that can be exacerbated or develop from an individual's response to emotional states.^4^ Some of these include psoriasis, atopic dermatitis, non‐specific dermatoses and pruritus (Table 2).^3^, ^4^, ^5^, ^6^, ^7^, ^8^, ^9^, ^10^, ^11^ These will all be discussed in specific paragraphs that follow the introduction, definitions, epidemiology and aetiology paragraphs.

**TABLE 1:** Some cutaneous somatic symptoms

Pruritus
Pain
Burning
Numbness
Soreness
Blotchiness
Paraesthesia
Unexplained purpura

**TABLE 2:** Skin diseases which may display somatic symptoms

Psoriasis and psoriatic arthritis
Atopic dermatitis (atopic eczema)
Pruritus
Nodular prurigo
Vulvodynia
Male genital dysaesthesia
Burning mouth syndrome
Urticaria and angioedema?

## 3 DEFINITION OF SOMATIC DISORDERS

Somatization is the phenomenon of experiencing bodily symptoms, most commonly pain and itch, in the absence of a biological cause.^1^, ^2^, ^12^ Somatization may occur with or without the existence of a dermatological disease.^1^, ^2^, ^12^ Somatic symptoms can be solely focussed on the skin, with pruritus or discomfort being the most prevalent,^3^ or with other symptoms including headache, back pain, exhaustion, gastrointestinal symptoms, chest pain, shortness of breath and paresthesiae.^12^ There is often a preoccupation with abnormal thoughts, feelings and behaviours.^12^, ^13^ This leads to significant stress and for the affected individual.^12^, ^13^ Disorders presenting with cutaneous somatic symptoms (Table 1), or with somatic overlay in established immunodermatoses (Table 2), frequently present in both primary and secondary care.^2^, ^14^

Somatic symptom disorders and somatic symptoms and related disorders have been newly categorized in the Diagnostic and Statistical Manual of Mental Disorders, fifth edition (DSM‐5) within the last 10 years (Table 3).^13^

**TABLE 3:** Somatization—diagnostic criteria

Diagnostic Criterion
One or more somatic symptoms that are distressing or result in significant disruption of daily life. Excessive thoughts, feelings, or behaviours related to the somatic symptoms or associated health concerns as manifested by at least one of the following: Disproportionate and persistent thoughts about the seriousness of one's symptoms. Persistently high level of anxiety about health or symptoms. Excessive time and energy devoted to these symptoms or health concerns. Although any one somatic symptom may not be continuously present, the state of being symptomatic is persistent (typically more than 6 months).
Specify if
**With predominant pain** (previously pain disorder): This specifier is for individuals whose somatic symptoms predominantly involve pain.
Specify if
**Persistent:** A persistent course is characterized by severe symptoms, marked impairment, and long duration (more than 6 months).
Specify current severity
**Mild:** Only one of the symptoms specified in Criterion B is fulfiled. **Moderate:** Two or more of the symptoms specified in Criterion B are fulfiled. **Severe:** Two or more of the symptoms specified in Criterion B are fulfiled, plus there are multiple somatic complaints (or one very severe somatic symptom).

*Source*: From the Diagnostic and Statistical Manual of Mental Disorders, Fifth Edition (Copyright ©2013), American Psychiatric Association. All Rights Reserved.

### 3.1 Epidemiology

The prevalence of somatic symptom disorder is uncertain because it is difficult to measure.^15^ Thus, the estimate in relation to somatic symptom disorder is obtained from literature from somatoform disorders.^13^, ^15^

Somatic symptom disorder in the general adult population can be approximated to 5%–7%.^16^ Females tend to present with somatic symptom disorder more often than males, with an estimated female‐to‐male ratio of 10:1.^17^ Other risk factors include low socioeconomic status, older age, fewer years of education.^13^, ^15^, ^17^, ^18^, ^19^ Increased severity of somatic symptom disorder is linked to childhood sexual abuse, concurrent physical or psychiatric illness and a history of substance abuse.^13^, ^15^, ^17^, ^18^, ^19^

In primary care about 20% of patients may present with somatic symptoms, in the absence of an organic medical condition.^17^

### 3.2 Aetiology

Somatic symptom and related illnesses can be caused by a variety of circumstances.^1^, ^13^, ^15^ Genetic and environmental vulnerability are all factors to consider.^1^, ^13^, ^15^ These include having an increased sensitivity to pain, childhood trauma and learning, and cultural or social norms that devalue and stigmatize psychological suffering in comparison to physical suffering.^1^, ^13^, ^15^ The consequences of such trauma in the absence of psychological insight can become overwhelming for the individual.^1^ The emotional symptoms from the psychological trauma manifests itself as somatic complaints referred to the skin.^1^ Emotional experiences, or emotional expression may be suppressed in individuals from non‐Western cultures, due to stigmata connected with psychiatric issues.^20^ Somatization is undoubtedly a global phenomenon.^21^, ^22^ However, its clinical presentation may be modified in significant ways by culture.^21^, ^22^ Genetic factors play a role in the predisposition to chronic pain and other bodily symptoms.^18^ However, no single gene has been linked with somatization specifically.^18^ Epigenetic mechanisms could explain how exposure to psychological trauma could lead to the development of subsequent cutaneous somatization.^22^, ^23^

## 4 SOMATIZATION AND PSORIASIS

The psychological implications of somatic disorders impact patients' quality‐of‐life.^5^ For example, psoriasis patients have increased levels of hypochondriasis, hysteria and other manifestations of somatization.^5^, ^6^, ^7^, ^8^, ^9^ Psychosomatic factors, such as stressful life experiences, a lack of social support and attachment instability, could explain why psoriasis patients have higher somatization rates.^2^ Both depression and suicidal ideation are more common in psoriasis patients, for example.^10^

Increasing psoriasis area and severity index scores correlate with increasing levels of somatization.^24^ Alexithymia, or the inability to describe one's emotions, positively correlates with somatization in patients with psoriasis, especially in women and also when the face, hands or genitals are involved.^25^, ^26^ Psoriatic arthritis (PsA) is a seronegative spondyloarthropathy characterized by skin lesions, dactylitis, and enthesitis.^27^ Patients with PsA often suffer from a number of psychosocial problems and also nonspecific symptoms early on in the course of the disease.^27^ They continue to experience progressive disease due to delays in diagnosis and treatment.^27^ Symptoms initially viewed as somatization could lead to undertreatment and promote psychological distress, poor coping, and negative patient‐provider relationships.^27^

### 4.1 Somatization and atopic dermatitis (eczema)

Depression, anxiety and somatization are all firmly and separately linked with atopic dermatitis (atopic eczema; AD) in both genders, when compared to mild AD.^11^, ^28^ Early treatment of the clinical dermatosis might reduce the probability of psychiatric problems.^11^, ^28^ Somatization is also positively correlated with AD and obsessive‐compulsive disorder in both genders.^28^

### 4.2 Somatization, pain and pruritus

Pain and itch sensations share common efferents in the peripheral nervous system.^3^ There is a decussation in the spinal cord and both sensations are perceived in distinct brain regions.^3^ Perhaps this is why pain, itch and ‘burning’ (itch combined with pain) are such common somatic symptoms and can occur together.^3^

Persistent somatoform pain disorders are characterized by severe pain, burning or tingling in the skin, either locally or more generally.^29^ General skin examination is usually normal.^29^ Some patients do experience additionally somatoform autonomic dysfunction.^29^ Where there is a cutaneous abnormality, it presents as flushing or sweating.^29^ This is often associated with transient dysaesthesia.^29^

Several chronic idiopathic mucocutaneous pain syndromes exist where patients may have associated psychosexual problems.^29^, ^30^ However, it is not clear whether either these psychological difficulties, or the physical symptoms occurs first.^29^, ^30^ Examples of such disorders include vulvodynia, which is unexplained burning and discomfort in the vulva, often exacerbated by intercourse.^29^, ^30^ Similarly, men may present with penodynia or scrotodynia, describing episodic genital burning pain on a background of low‐grade ache.^29^, ^30^ Local erythema is often found too.^29^ Burning mouth syndrome is characterized by an idiopathic burning pain and dry mouth, with no clinically apparent changes.^29^, ^30^, ^31^ It can present to a variety of health professionals including dermatologists.^29^, ^30^, ^31^ It is another form of chronic pain disorder.^29^, ^30^, ^31^

Functional pruritus occurs where psychological factors play an evident role in the triggering, intensity, aggravation or persistence of the itch.^3^, ^29^, ^30^ On physical examination, no evidence of dermatoses will usually be present.^3^, ^29^, ^30^ However, excoriations may occur, often with a ‘butterfly sign’ of completely normal skin over the mid‐upper back.^3^, ^29^, ^30^

Multiple chemical sensitivity and idiopathic environmental intolerance (MCS/IEI) is a term for a range of symptoms in various organs, including the skin.^29^ Symptoms are triggered by exposure to substances at levels would not normally affect the general public.^29^ In addition, there are no positive laboratory investigations.^29^ Symptoms can include nasal stuffiness, fatigue, difficulty concentrating, amnesia and itch.^29^

### 4.3 Urticaria, angioedema, anaphylaxis and somatization

Urticaria, angioedema and anaphylactoid symptoms can present via somatization in response to traumatic memories or flash backs.^1^, ^2^, ^29^, ^30^ Angioedema of the mouth or tongue with no clear physical cause may be linked to flashbacks of oral sexual abuse.^1^ Clearly, conventional urticaria and angioedema can also be linked to stress and this is where the boundaries with somatization are blurred.^32^

Although there has been little that has been published about somatoform idiopathic anaphylaxis recently, it will still present to tertiary care allergy clinics.^29^ Patients experience symptoms simulating anaphylaxis.^29^ They may complain of swelling sensations within the oropharynx or laryngopharynx, without any objective signs being seen.^29^

### 4.4 Other cutaneous presentations and associations with somatization

There are other cutaneous associations with somatization. Somatization has been reported in women particularly, with non‐melanoma skin cancer.^33^ Patients with nodular prurigo have comparable levels of somatization to patients with psoriasis.^34^ Patients with somatization can imagine that normal or variations of normal in the skin have some pathological significance.^35^ This is distinct from body dysmorphic disorder, which is a variation of depression, where the individual is unhappy with their appearance.^36^ It is also distinct from delusional infestation too, where there is no insight into the perceived cause of the problem.^37^ For example, a normal cutaneous finding, such as insect bites, would provoke simple annoyance in a normal individual.^35^ However, this is perceived as a physical and psychological threat to the individual with somatization.^35^ This could then present with other unrelated physical symptoms, such as paraesthesiae.^35^ Rosacea and cutaneous flushing can also be associated with somatic symptoms too.^35^ Gardner‐Diamond syndrome (GDS) is the clinical picture of painful cutaneous and mucosal ecchymoses in women.^38^ The exact cause of the disease is unclear and some consider it to be a somatoform disorder.^38^ More recently, antibodies against phosphatidylserine in erythrocyte stroma have been detected, perhaps causing immune complexes and complement activation in GDS, suggest a possible autoimmune pathogenesis instead.^38^

## 5 DIFFERENTIAL DIAGNOSIS

The differential diagnosis for patients with suspected somatic symptom disorder may include depression, panic disorder, generalized anxiety, substance abuse, psychiatric syndromes of unclear aetiology and non‐psychiatric medical conditions, including neurological disorders.^17^ All of these must be ruled out as an explanation for the symptoms.^17^

### 5.1 Treatment

It is important to manage any underlying skin disease first (Table 2).^39^ Treatment options for treating somatization can include psychotherapy as cognitive behavioural therapy (CBT) and mindfulness‐based therapy (MBT).^17^, ^23^ Pharmacotherapy is also an option for treatment of somatization.^17^, ^23^ This would include treatment with antidepressants, antiepileptics, antipsychotics and herbal products such as St. John's wort.^17^, ^23^ Management may involve treating the patient with both CBT (or related psychotherapy approaches) and an antidepressant, especially if there is associated anxiety and depression (Table 4).^2^, ^43^, ^44^ CBT can be used as first‐line intervention for somatic symptoms or in adjunctive therapy for patients that fail another treatment options such as psychotropic medication or other forms of psychotherapy.^43^ CBT may be more effective at treating physical somatization symptoms, than coexisting depressive or anxiety disorders.^43^ MBT is an 8‐week psychological treatment course.^44^ It has shown to lead to significant improvement in symptoms of anxiety and depression.^44^ A recent study showed that MBT, in addition to the antidepressant venlafaxine, in patients with somatization can significantly reduced the severity of the psychological and physical symptoms in these patients, compared with venlafaxine treatment alone.^44^ Electroconvulsive therapy has been used in the treatment of somatization, with comorbid mood disorders and skin disease, although it would very much be a last resort therapeutically.^43^

**TABLE 4:** Treatment options for somatic symptoms

Modality	Findings
Cognitive behaviour therapy (CBT)^13^, ^40^, ^41^	*Useful in psychological conditions where the specific dermatologic condition is triggered or exacerbated in the presence of a heightened emotional state or stressful life events.*
	*Effective for treatment of somatization and medically unexplained symptoms.*
	*CBT could be used either as a first‐line intervention for persistent somatic symptoms or as adjunctive therapy for patients who fail other treatment strategies.*
Mindfulness therapy/based stress reduction (MBT)^42^	*Reduces of somatic symptoms as well as the severity and number of physical symptoms can be obtained when MBT accompanied by an serotonin‐norepinephrine reuptake inhibitors (SNRI) (venlafaxine).*
Pharmacotherapy^2^, ^3^	Selective serotonin reuptake inhibitor, SNRI's, tricyclic antidepressants.
	*psychiatric* *and neurological comorbidities should be evaluated and excluded prior to initiation of treatment.*

### 5.2 Prognosis

Somatic symptoms can be chronic, with waxing and waning features.^19^ Spontaneous recovery can occur, with around 50%–75% of patients with medically unexplained symptoms improving, whereas 10%–30% deteriorate.^19^

## 6 CONCLUSION

In conclusion, somatization in dermatology is a continued evolving intention to understand the mind and skin connection. Numerous skin conditions may arise because of psychological disturbances. Somatic symptoms can be solely focussed on the skin (Table 1), with pruritus or discomfort being the most prevalent, or they can be accompanied by other symptoms, including headache, back pain, exhaustion, gastrointestinal symptoms, chest pain, shortness of breath, and paresthesiae. The presentation of somatization in a dermatological patient has been challenging with the initial management, diagnosis, and treatment as the field of psychodermatology continues to expand in research and clinical relevance (Table 2). The use of both psychotherapy and antidepressants will remain important, as well as the management of any underlying skin disease (Table 4).

## AUTHOR CONTRIBUTIONS

**George W. M. Millington**: Conceptualization (equal); Data curation (equal); Formal analysis (equal); Funding acquisition (equal); Investigation (equal); Methodology (equal); Project administration (equal); Resources (equal); Supervision (equal); Writing – review & editing (equal). **Morinola T. Shobajo**: Conceptualization (equal); Writing – original draft (equal). **James Wall**: Funding acquisition (equal); Resources (equal). **Mohammad Jafferany**: Conceptualization (equal); Data curation (equal); Formal analysis (equal); Investigation (equal); Project administration (equal); Supervision (equal); Writing – original draft (equal); Writing – review & editing (equal).

## CONFLICT OF INTEREST

George W. M. Millington is the Editor in Chief of Skin Health and Disease.

## Data Availability

As a review article, there are no original data.
